# Nanoemulsion based Intranasal Delivery of Antimigraine Drugs for Nose to Brain Targeting

**Published:** 2009

**Authors:** R. S. Bhanushali, M. M. Gatne, R. V. Gaikwad, A. N. Bajaj, M. A. Morde

**Affiliations:** C. U. Shah College of Pharmacy, S. N. D. T. Women's University, Juhu Campus, Santacruz (West), Mumbai-400 049, India; 1 Bombay Veterinary College, Parel, Mumbai-400 012, India; 2 Department of Nuclear Medicine, Bombay veterinary College, Parel, Mumbai-400 012, India

**Keywords:** Intranasal nanoemulsions, intranasal mucoadhesive gels, brain-targeting, rizatriptan benzoate

## Abstract

The objective of this study was to develop intranasal nanoemulsion and gel formulations for rizatriptan benzoate for prolonged action. Nanoemulsion formulations were prepared by constructing pseudo-ternary phase diagrams using lipophilic and hydrophilic surfactants and water. Various mucoadhesive agents were tried out to form thermo-triggered mucoadhesive nanoemulsions. Mucoadhesive gel formulations of rizatriptan were prepared using different ratios of HPMC and Carbopol 980. Comparative evaluation of intranasal nanoemulsions and intranasal mucoadhesive gels indicated that greater brain-targeting could be achieved with nanoemulsions.

Migraine is a neurologic disease, associated with throbbing intense headache in one half of the head. Rizatriptan is 5HT 1B/1D receptor agonist with an oral bioavailability of 40% and half life of 2-3 h. To increase the bioavailability and brain tissue deposition for antimigraine therapy, intranasal delivery systems were developed. Intranasal administration offers a practical, noninvasive, alternative mode for drug targeting to the brain. Absorption of drug at the olfactory region of nose provides potential for drugs to be directly delivered to the CNS. However, one of the problems associated with nasal delivery is lower retention time of solution in nasal cavity (15 min) resulting in lower bioavailability. Hence, the objective of this study was to develop intranasal nanoemulsion and gel formulations for rizatriptan benzoate for prolonged action, controlled release and direct targeting of drug to the brain tissues.

## MATERIALS AND METHODS

Rizatriptan benzoate (Jiangsu, China), Labrafil and Transcutol P (Colorcon India Pvt Ltd.), Cremophor RH 40, PF127 and PF68 (BASF Pvt Ltd.), Carbopol 980 (Noveon India), HPMC (Signet Chemicals Ltd).

### Preparation and evaluation of thermo-triggered mucoadhesive nanoemulsions:

**Fig. 1:**
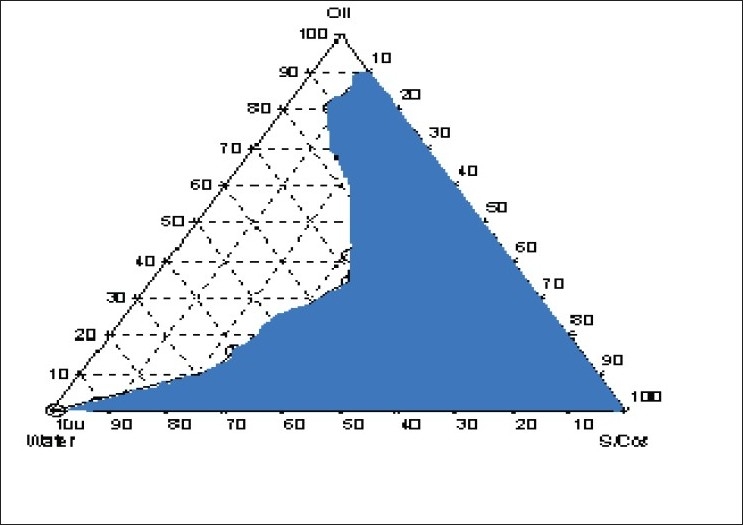
Phase diagram of optimized nanoemulsion formulation

Nanoemulsion formulations were prepared by constructing pseudo-ternary phase diagrams using lipophilic and hydrophilic surfactants and water (fig. 1). Varying ratios of pluronics PF 127/PF 68 were added to form thermoreversible nanoemulsions. Various mucoadhesive agents were tried out to form thermo-triggered mucoadhesive nanoemulsions. Nanoemulsions were evaluated for clarity, viscosity, globule size, mucoadhesive strength and drug content (Table 1)

**TABLE 1:** EVALUATION OF DEVELOPED INTRANASAL FORMULATIONS

Tests	Thermoreversible Nanoemulsion	Mucoadhesive Gel
Clarity	Clear 99% transmittance	Transparent
pH	6.0-6.5	7.0-7.2
Viscosity	700 cps	50000 cps
Stability	Stable by centrifugation	Stable
Particle Size	20-120 nm	--
Drug Content	98.6%	99.13%

### Preparation and evaluation of intranasal mucoadhesive gels:

**Fig. 2:**
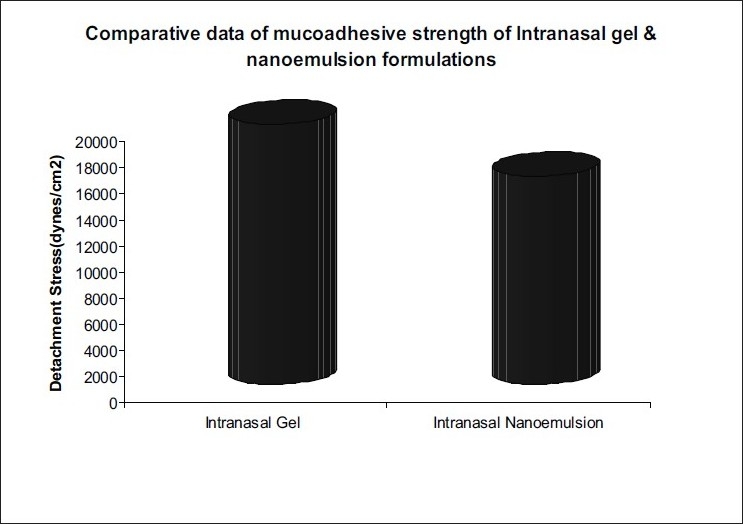
Mucoadhesion effect of developed formulations

Mucoadhesive gel formulations of rizatriptan were prepared using different ratios of HPMC and Carbopol 980. The pH of the gel was adjusted to 7.0±0.2 with the help of triethanolamine. Sodium metabisulphite (SMB) and methyl paraben were added as antioxidant and preservative, respectively. Penetration enhancers like Lutrol F68 and sodium lauryl sulfate were tried out. Mucoadhesive gels were evaluated for appearance, pH, viscosity, consistency, mucoadhesive strength (fig. 2) and drug content.

### *Ex vivo* diffusion studies:

**Fig. 3:**
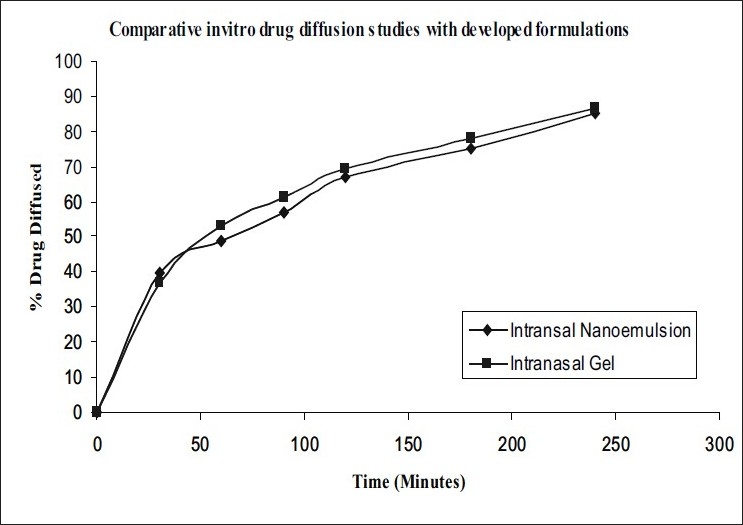
Comparative *in vitro* diffusion profiles Comparative *in vitro* diffusion profiles of (–◆–) intranasal nanoemulsion and (–●–) intranasal gels

Diffusion kinetics were carried out using sheep nasal mucosa in phosphate buffer (PB) pH 6.4 for a period of 4 h using Keshary-Chein apparatus (fig. 3). The drug content was measured in PB using developed and validated reverse phase HPLC method at λmax 224nm.

### Nasal mucosal irritation Studies:

**Fig. 4:**
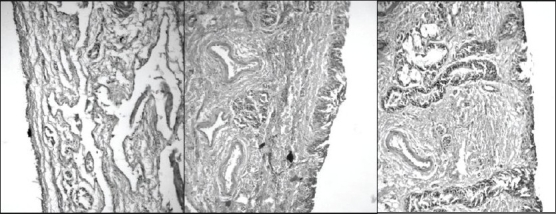
Histopathological evaluation of sheep nasal mucosa for assessing irritation potential

Irritation potential of the developed formulations was assessed by histopathological studies on stained sheep nasal mucosa (fig. 4).

### *In vivo* brain targeting potential:

The amount of drug in the homogenized brain tissue of Wistar rats was detected and analyzed using developed and validated reverse phase HPLC method. The rizatriptan deposition in brain tissue from the developed intranasal formulations was compared with that obtained after IV administration (Table 2 and 3) as per the protocol approved by Animal Ethical Committee.

**TABLE 2:** COMPARATIVE DATA OF BRAIN TARGETING EFFICIENCY WITH NANOEMULSION AND GEL FORMULATIONS

Formulations	Drug targeting Efficiency (%DTE)	Direct Nose-to-Brain transport (%DTP)
Intranasal Gel	289 ± 3	20.64
Intranasal Nanoemulsion	106 ± 2	63.20

**TABLE 3:** PHARMACOKINETIC PARAMETERS FOLLOWING I.V AND INTRANASAL ADMINISTRATION OF RB-LOADED TTM NANOEMULSION AND GEL FORMULATIONS

Parameters	Route	Plasma	Brain
C_max_ (ng/ml g)	i.n.(nano)	7806.137±613.2^a^	2954±255.6
	i.n.(gel)	7702.915±657.1	670±60.7
	i.v.	7847.500±657.1	597±60.7
T_max_ (min)	i.n.(nano)	15	30
	i.n.(gel)	15	30
	i.v.	15	30
AUC0→240	i.n.(nano)	1707290±2789	302520±2475
(ng min/ml g)	i.n.(gel)	1732516±3935	115000±3821
	i.v.	1800538±3134	109627.5±3014
Ratio of AUCi.n./AUCi.v.(%)	i.n.(nano)	94.84^b^	275.95
	i.n. (gel)	96.22^b^	

i.v.: RB solution; i.n. (nano) RB nanoemulsion; i.n. (gel) RB-gel

a Mean±SD (n=3-4)

b bioavailability.

## RESULTS AND DISCUSSION

Nanoemulsions with S/Cos (3:1) and gels were transparent, stable with high drug incorporation. *Ex vivo* diffusion studies of the developed formulations gave controlled release with 86% in 4 h. Both the formulations exhibited bioadhesion. Brain targeting of IN nanoemulsions (AUC=302.52 μg min/g) was higher as compared to IN Gels (AUC=115 μg min/g) and IV administration (AUC=109.63 μg min/g) of the drug. Thus brain targeting through intranasal delivery has a potential for treatment of migraine.

## References

[1] Behl CR, Pimplaskar HK, Sileno AP, DeMeireles J, Romeo VD (1998). Effects of physiochemical properties and other factors on systemic nasal drug delivery. Adv Drug Delivery Rev.

